# Improving Sustainable Financing for Universal Health Coverage in Bhutan: Exploring Policy Options and Financial Strategies

**DOI:** 10.1002/puh2.216

**Published:** 2024-07-08

**Authors:** Ugyen Tshering, Jayendra Sharma, Dorji Tshering, Tandin Dendup

**Affiliations:** ^1^ Department of Public Health Ministry of Health Thimphu Bhutan; ^2^ Center for Health Policy Thimphu Bhutan; ^3^ Phuentsholing General Hospital Phuntsholing Bhutan; ^4^ Policy and Planning Division Ministry of Health Thimphu Bhutan

**Keywords:** Bhutan, health financing, health policy, health system strengthening, policy options, sustainable financing, universal health coverage

## Abstract

Deeply rooted in its developmental philosophy of gross national happiness (GNH), Bhutan's healthcare system strives towards achieving a shared goal of universal health coverage (UHC). Despite being primarily financed by the government, the health system faces a plethora of challenges. To overcome these hurdles and achieve UHC goals, expanding the fiscal space for health and improving operational efficiency are crucial. This article aims to address Bhutan's evolving healthcare landscape and advance the achievement of UHC through two policy options. The first policy option focuses on the dual objective of improving health outcomes and promoting financial sustainability by leveraging health taxes, whereas the second option emphasizes reinforcing a systematic health technology assessment (HTA) in the Bhutanese health system. First, drawing lessons from global experiences, the policy brief recommends leveraging health taxes to reduce societal and healthcare costs and enhance financial sustainability in the health sector. Considering Bhutan's high prevalence of tobacco and alcohol consumption, and taking opportunity from the ongoing Goods and Services Tax (GST) reform, continued advocacy on health taxes is essential, and soft earmarking the health taxes may be considered to finance a broader array of public health programmes, particularly focusing on the promotion of healthy lifestyle, health screening and outreach public health activities. Second, the integration of HTA into policymaking and decision‐making processes is essential for effective resource allocation in UHC. Nurturing and strengthening the existing HTA governance structure under the Ministry of Health (MoH) and establishing a dedicated multidisciplinary HTA Committee will ensure informed decision‐making and resource optimization. HTA evidence should inform the revision of health service standards, clinical guidelines development, procurement decisions and healthcare priorities. These policy options can assist the country in improving financial sustainability, enhancing effective resource allocation and utilization and improving healthcare delivery, aligning with its vision of GNH and ultimately accelerating progress towards achieving UHC.

## Graphical abstract

This study examines Bhutan's unique healthcare system, facing challenges despite government funding. To achieve universal health coverage (UHC), it proposes two policy options:

1. Leverage health taxes (on tobacco/alcohol) to both improve public health and raise funds for the healthcare system.

2. Integrate health technology assessment (HTA) into decision‐making to ensure cost‐effectiveness and optimize resource allocation.

By implementing these policies, Bhutan can improve financial sustainability, allocate resources effectively and accelerate UHC progress while staying true to its gross national happiness philosophy.

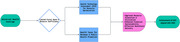

**Abbreviations**
CaHE: catastrophic health expenditure
FY: financial year
GDP: gross domestic product
GGE: general government expenditure
GNH: gross national happiness
GST: Goods and Services Tax
HITAD: Health Intervention & Technology Assessment Division
HITAP: Health Intervention & Technology Assessment
HTA: health technology assessment
MoF: Ministry of Finance
MoH: Ministry of Health Programme, Thailand
THE: total health expenditure
UHC: universal health coverage

## 1 Introduction

Access to quality healthcare services is a fundamental right of every human being, and achieving universal health coverage (UHC) is a shared global target for achieving the Sustainable Development Goals (SDGs). In Bhutan, the pursuit of UHC is deeply embedded in the nation's unique developmental philosophy of gross national happiness (GNH) [1]. GNH emphasizes holistic well‐being and the interdependence of social, economic and environmental factors. Further, Article 9 Section 21 of the Constitution of the Kingdom of Bhutan mandates the state to provide free access to basic public health services [2]. This provision of free healthcare services to all citizens is a notable aspect of Bhutan's healthcare system.

**FIGURE 1:**
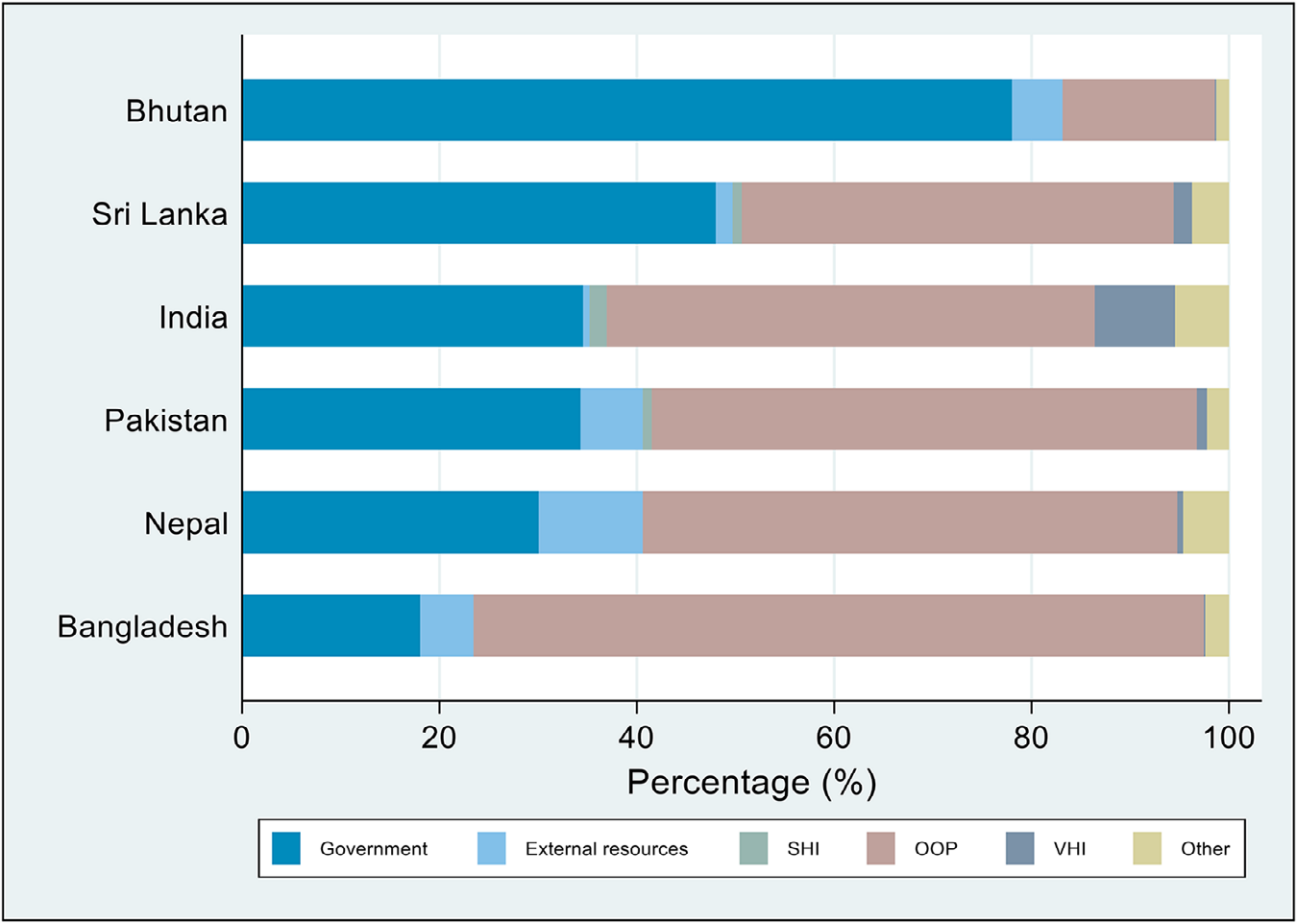
Sources of healthcare finance in South Asian countries, 2020. *Source*: Global Health Expenditure Database [4].

The healthcare system is predominantly financed by the government in Bhutan (Figure 1). Private sector engagement in healthcare is limited to diagnostic centres, whereas the health insurance system is just restricted to outbound travellers and certain groups of tourists. In 2021, the proportion of gross domestic product (GDP) allocated to total health expenditure stood at 4% and 4.5% during the financial years (FY) 2018–2019 and 2019–2020, respectively [3]. In these 2 years, the government's portion to the current health expenditure (CHE) was 70.2% and 73.4%, whereas households’ out‐of‐pocket (OOP) expenditures constituted 18% and 15.4%, respectively.

In 2017, catastrophic health expenditure (CaHE) at the 40% threshold was estimated at 0.51%, impoverishment at 0.32% and further impoverishment at 1.93%, resulting in a total financial hardship of 2.55%. Rural dwellers and poorer households experienced a heavier burden of financial hardship [5]. A study using data from 2012 estimated the incidence of CaHE at 4.06% with a 10% household budget threshold [6]. Although these figures are lower than those of many other countries in the WHO Southeast Asian region [6], a significant proportion of households in Bhutan still face financial hardship despite having free healthcare.

Issues related to inefficiency of health expenditure and ineffectiveness in resource utilization are also widely reported [7, 8, 9]. The National Referral Hospital lost Bhutanese Ngultrum (Nu.) 117.9 million (US$1.4 million) for radiation therapy services that did not provide any benefit to patients over the past 5 years [10]. Conducting health technology assessment (HTA) prior to the rolling out of such services could have prevented this. Moreover, Bhutan's UHC effective coverage index for 2020 was 51, which is comparatively lower than that of several neighbouring countries in the region with similar or lower level of investment in health (Table 1) [11]. Considering its level of health expenditure, Bhutan has room for improvement in terms of efficiency and quality to achieve better health outcomes [8, 12].

**TABLE 1:** Comparing the universal health coverage (UHC) effective coverage index to health spending per capita in South Asian countries.

Countries	UHC effective coverage index	Health spending per capita (in US$)
Bhutan	51	116
Nepal	47	53.25
Bangladesh	54	45.86
Sri Lanka	66	161
India	47	64
Pakistan	39	39

*Source*: Lozano et al. [11].

**FIGURE 2:**
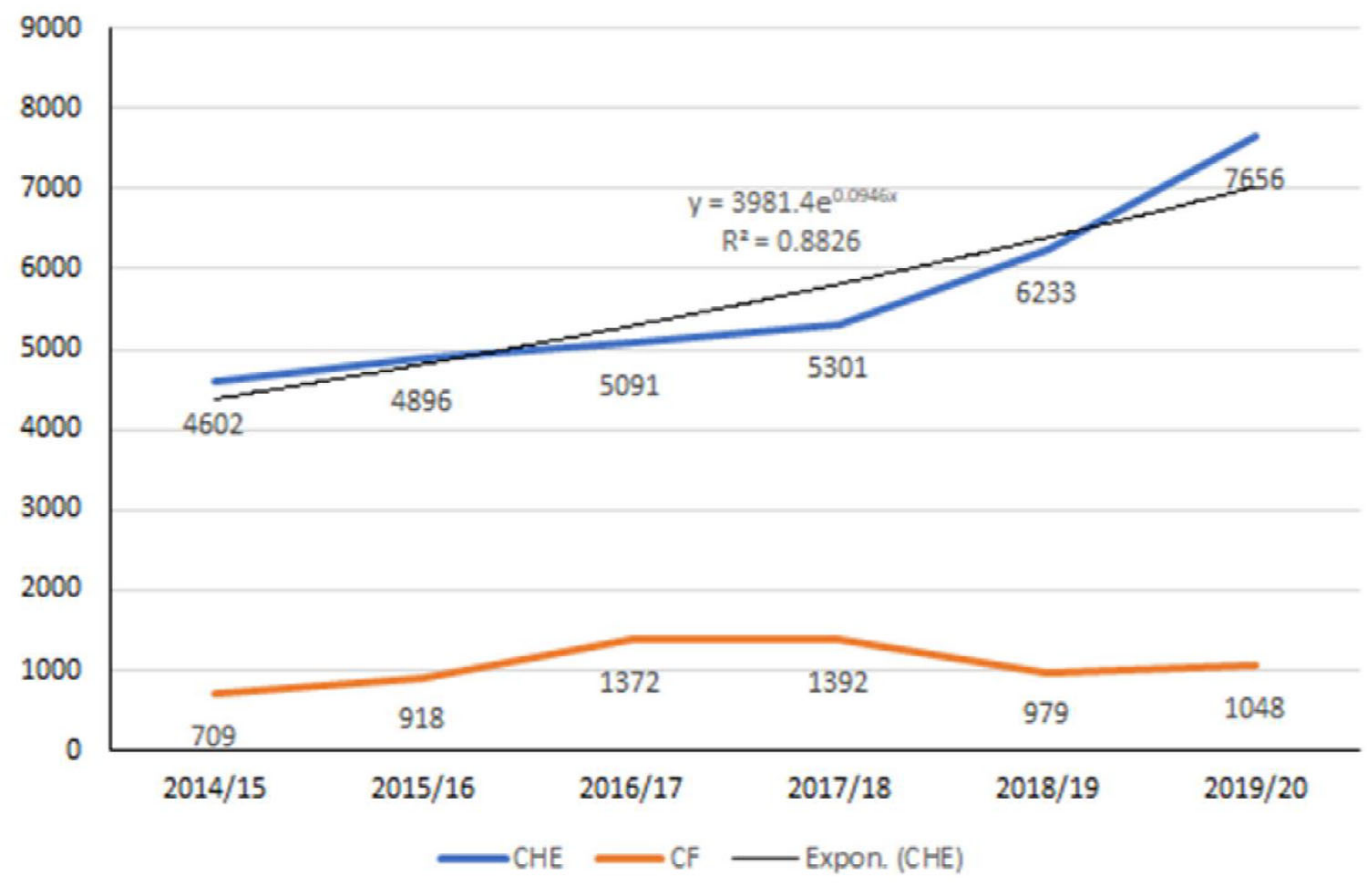
Trends in CHE and capital formation between FY 2014/15 and FY 2019/20. CHE, current health expenditure; FY, financial year. *Source*: NHA [13].

The cost of providing free healthcare services has consistently increased over the years. The CHE increased by 17% in 2018–2019 compared to 2017–2018 and by 22% in 2019–2020 from the previous FY (Figure 2) [13]. The rise in cost has been exacerbated by the expansion of essential health services nationwide, growing public expectations, patient referrals abroad, technological advancement and the ongoing shift in epidemiology, demography and economy [2, 7, 8]. Hence, in the backdrop of all these contributing factors, the government is encountering challenges in finding a sustainable solution for health financing [3, 15]. This calls for the need to explore innovative and alternative financing strategies for long‐term sustainability [12].

This article aims to examine additional health financing mechanisms that can enhance fiscal space for health, thereby contributing to the achievement of UHC in Bhutan.

## 2 Policy Options

The following two policy options are carefully considered by examining both global evidence and the unique local context of Bhutan. By incorporating global evidence, the policy options benefit from the lessons learned and best practices observed in other countries. Moreover, the policy options are tailored to the needs of the country, considering the existing healthcare system and economic landscape. A synopsis of the comparison of two policy options with various indicators is given in Table 2.

**TABLE 2:** Comparisons of two policy options on enhancing sustainable health financing for universal health coverage (UHC) in Bhutan.

**Policy options**	**Leverage health taxes to enhance both health outcomes and financial sustainability**	**Reinforcing health technology assessment (HTA) for optimal resource allocation**
**Description**	The policy aims to discourage harmful substance consumption, generate additional revenue to promote public health by reducing consumption of unhealthy products and ensure additional funding for healthcare system	This policy focuses on reinforcing HTA as a vital tool for informed decision‐making on resource allocation. Evaluating technology effectiveness ensures value for money, efficient allocation and maximize health benefits towards achieving UHC
**Advantages**	‐ Improved public health outcome – decrease in NCDs ‐ Enhance financial sustainability through additional funding source ‐ Adoption of healthier lifestyle choices through behavioural change	‐ Evidence‐based decision‐making in resource allocation ‐ Efficient use of limited healthcare budget ‐ Cost containment, thereby controlling healthcare costs ‐ Equitable access to healthcare services
**Disadvantages**	‐ Economic implications and potential job losses ‐ Growth of black market and smuggling activities ‐ Equity concerns – burden on lower incomes ‐ Risk of reduction in the budget from main funding pool	‐ Requirement of extensive resources like expertise, data, time and integration ‐ Potential resistance from stakeholders ‐ Delays in access to new healthcare technologies
**Stakeholders**	Parliament, MoH, MoF, alcohol industries, tobacco distributors and retailers, consumers, CSOs, media, international institutions, industry associations	MoH, university, healthcare providers, policymakers, private sectors (like pharmaceutical companies), HITAP (int. partner), patients, media
**Acceptability**	Pro – MoH, MoF, WHO, World Bank Mixed – Parliament, consumers, CSOs, media Against – alcohol and tobacco industries, distributors and retailers	Pro – MoH, university, HITAP, patients Mixed – healthcare providers, policymakers, media Against – private sector

Abbreviations: HITAP, Health Intervention and Technology Assessment Program, Thailand; MoF, Ministry of Finance; MoH, Ministry of Health.

### 2.1 Leverage Health Taxes to Enhance Both Health Outcomes and Financial Sustainability

The government's domestic revenue heavily relies on a combination of taxes (66%) and non‐tax revenue sources (34%) [16]. Taxes from income, profit and capital gains and other taxes (mainly royalties) contribute to the largest share of the total revenue. Hydropower is one of the significant contributors to the revenue; however, it could be highly volatile down the road. Additionally, the government's current account deficit hit an all‐time high of 33% of GDP in FY 2021–2022 [17], suggesting that there could be implications for domestic resource allocation, including healthcare.

The government's allocation to fiscal space for health was 10% of the general government expenditure, which corresponds to 3% of GDP [4]. Considering this, the current allocation suggests limited scope for reprioritization or increasing the budgetary share for health. Moreover, there has been a decline in external funding for the health sector over the years [13, 14, 15]. As Bhutan graduates from the United Nations Least Developed Country (LDC) category in 2023 [18], these resources are expected to decline further. The social health insurance mechanism may not be feasible in Bhutan considering the small base of its formal sector [14]. Therefore, it is essential to explore additional and appropriate sources to expand the fiscal space for health [8, 12, 15, 19].

In 2021, alcohol liver disease ranked as the leading cause of death in Bhutan, whereas respiratory diseases were among the top five causes of mortality. These statistics highlight the substantial burden that the consumption of harmful substances imposes on health and well‐being. The prevalence of tobacco use among adults was 24.8 [20], whereas the prevalence of current alcohol use was 30.6% [21]. The annual cost for alcohol‐related treatment in the country was approximately Nu. 29–30 million (US$350,000), which accounts for 1.84% of CHE [22]. In 2014, it was reported that the socio‐economic cost of alcohol reached Nu. 5 billion (US$70 million), which was four times higher than the revenue generated by alcohol sales [23]. Overall, 69% of all deaths in 2016 were caused by non‐communicable diseases [24], which represents a growing epidemic necessitating a multifaceted response, particularly targeting unhealthy diets and lifestyle.

One promising measure that could accrue both public health benefits as well as generate supplementary resources for healthcare is health taxes, irrespective of whether they are earmarked specifically for the health sector. Health taxes are taxes that are imposed on the consumption of goods that have negative health impacts, such as tobacco, alcohol, sugar‐sweetened beverages and other unhealthy products. By taxing products that are harmful to public health, it supports the healthcare system by reducing societal and healthcare costs and increasing fiscal space [25]. Currently, there is no system of earmarked taxes specifically allocated to the health sector in Bhutan. All sin taxes on alcohol and tobacco products contribute to the general revenue as sales tax. The only approach close to earmarked tax would be the mandatory health contribution of 1% from basic salary in the formal sector since 2014. This fund directly gets transferred to the Bhutan Health Trust Fund (BHTF) pool. BHTF is an organization dedicated to ensuring a sustainable financing mechanism to support essential medicines and critical vaccines for the country. The health contribution for FY 2021–2022 was Nu. 302.72 million (US$3630,000), which amounts to 0.8% of the total revenue for the year [16].

There are several arguments for and against the earmarking of health taxes, and results seem to depend on political priorities, context‐specific challenges and budget process. Although earmarking has helped countries advance and sustain a national health priority, in several contexts, it is unlikely to bring in prioritization and efficiencies because budgets are fungible and earmarking could introduce rigidities and inefficiencies. ‘Soft’ earmarking with broader expenditure purposes, more flexible movement of finance and aligned to the national budget system is preferred [25]. Irrespective of the earmarking debate, health taxes are critical both for their potential impact on health outcomes and their revenue‐raising potential.

After the introduction of the Sin Tax Reform Act in 2012, the Philippines was able to witness a 15.4% reduction in smoking prevalence from 2008 to 2018 [26]. In 2013, the total collection of sin tax from tobacco and alcohol was US$1.57 billion and US$737.7 million, respectively. Excise taxes accounted for 53% of cigarette retail prices on average [27], which is below the WHO target of at least 70% of retail prices [28]. This tax reform led to an exceptional year‐over‐year budget increase for the health sector [27, 29]. Success on earmarked health taxes in Thailand reveals that openness and the capacity to demonstrate health gains from health taxes are crucial to gain public trust and support [30]. The use of tobacco tax as an effective strategy to tackle the tobacco epidemic has also gained support in many other Southeast Asian countries [31]. In the context of alcohol, it is reported that 133,000 lives could be potentially saved annually by implementing a minimum level of 15% tax on the retail price per unit of alcohol [32, 33].

As an effort to raise revenue and address tax leakages, the government adopted the Goods and Services Tax (GST) Amendment Bill in 2022. However, the implementation of GST had to be deferred to 2024 due to unfavourable economic situations and the lack of operational readiness of the tax system [34]. Once implemented, a GST of flat 7% will replace all indirect taxes, such as sales tax, customs and excise duty. It also entails the imposition of a 20% Excise Equalisation Tax on sugar‐sweetened beverages and other unhealthy commodities, along with a 100% tax on alcohol and tobacco products [23, 34]. Currently, all health taxes contribute to the general revenue of the government as taxes and duties. This is the opportune moment for the Ministry of Health (MoH) to (i) continue to place health taxes on the radar of the Ministry of Finance and advocate for its periodic update and (ii) consider soft earmarking the health taxes (GST on tobacco, alcohol and sugar‐sweetened beverages) to finance a broader array of public health programmes, particularly focusing on the promotion of healthy lifestyle, health screening and outreach public health activities.

### 2.2 Reinforcing HTA for Optimal Resource Allocation in UHC

HTA is a multidisciplinary process that evaluates the value of health technologies to inform decision‐making and promote an equitable, efficient and high‐quality health system [35]. Health technologies include interventions, such as diagnostic tests, devices, medicines, vaccines, procedures, programmes and systems that are designed for healthcare purposes. Although the HTA unit in Bhutan was established in 2009 [36, 37], its growth has been impeded due to limited availability of resources and technical capacity and poor buy‐in among stakeholders [38, 39]. Most often, the importance of HTA is recognized during donor phase‐outs, discussions on the sustainability of vertical programmes and instances of unsuccessful health technology implementation. Therefore, to ensure the sustainability of UHC, a strong emphasis on robust HTA is imperative.

In Thailand, HTA has not only improved cost‐effectiveness, minimized wasteful spending on interventions and promoted evidence‐based healthcare decision‐making, but also protected households from catastrophic health expenses [40, 41]. In 2016, Thailand achieved significant cost reductions in antiretroviral drugs, intraocular cataract lenses, erythropoietin‐stimulating agents and coronary stents, resulting in estimated savings of US$257 million for the health sector [42]. In Myanmar, HTA played a crucial role in adopting the policy for using vouchers as a financing approach to facilitate access to mother‐and‐child health services. The Philippines has adopted HTA as a tool for the development of national formulary since 2013 [36]. In 2016, Vietnam utilized HTA to reform the benefits package of high‐cost medicines and medical devices under the Vietnam Social Security Scheme. This reform achieved estimated annual savings of 3335 billion VND (US$147 million) by eliminating inappropriate use without compromising health outcomes [36].

A study highlighted the importance of delivering services at the lowest level of health facilities in Bhutan for sustainability and cost‐effectiveness [43]. However, managing service demand and ensuring appropriate patient referral remains a challenge. The current allocation of health expenditure, with a larger share dedicated to curative care (54%) compared to preventive care (14%) [13], indicates patients’ preference for higher level hospitals. Enhancing access to primary healthcare and implementing a reliable referral system can lead to appropriate service utilization and reduced OOP expenses for households [40]. To achieve efficiency gains, adapting staffing to workload is crucial for both cost‐effectiveness and reduced waiting times [13]. Incorporating these findings into HTA processes can inform evidence‐based decision‐making and optimize resource allocation for improved healthcare delivery.

There are promising steps made on HTA that need to be nurtured. A process guideline has been established that provides an outline of steps and processes in the HTA [25]. Assessments of pneumococcal conjugate vaccines [44] and rotavirus vaccination [45] have significantly helped decision‐making for immunization in the country. However, the processes and institutions are still evolving and need to be nurtured and significantly strengthened.

In view of the above, the Health Intervention & Technology Assessment Division (HITAD) under MoH should be empowered for effective decision‐making and resource allocation. HITAD's in‐house technical capacity should be strengthened to ensure effective oversight of HTA activities nationwide and conduct rigorous and evidence‐based HTAs [39]. HTAs should be conducted on a need basis, and the current practice of limiting HTAs to two to three topics a year should be done away with [37, 38]. Stakeholders and public participation in identifying HTA topics will ensure that HTA evaluations address the needs of all stakeholders [36, 38, 39], and this should be integrated into the current HTA norms and practices. HITAD should strengthen institutional linkage and collaborate with international HTA networks such as the UK National Institute for Health and Care Excellence (NICE) and Thailand's Health Intervention and Technology Assessment Program (HITAP), to name a few. It can help enhance Bhutan's HTA capabilities, provide access to global best practices and facilitate the sharing of resources and evidence. Moreover, engaging local universities in HTA tasks can contribute to policy and capacity building, as seen in Vietnam [36].

Table 3 outlines various challenges and opportunities associated with both policy options.

**TABLE 3:** Challenges and opportunities in the implementation of two policy options.

**Policy options**	**Leverage health taxes to enhance both health outcomes and financial sustainability**	**Reinforcing health technology assessment (HTA) for optimal resource allocation**
**Obstacles/Challenges**	Opposition from stakeholders like manufacturers or distributors or retailers of products Compliance and enforcement issues like tax evasion Mixed perception and acceptance from public – perceive it as an additional financial burden Need for the new legal framework and administrative system	Absence of a governing body and strategic plan for HTA systems development Insufficient budget support for HTA work Linking HTA to policymaking and stakeholders buy‐in Lack of technical capacity and retention of HTA researchers at MoH Rise in advanced biotechnologies
**Facilitators/Opportunities**	Reduce consumption of harmful products and improve public health outcomes Increase revenue for healthcare financing and potential reduction in healthcare costs Adopt global evidence and best practices for policy support Engage relevant stakeholders in the policy development process Raise awareness about benefits and rationale behind these health taxes Establish clear guidelines and mechanisms for tax collection	Establish HTA committee to oversee HTA's development and implementation Establish a strong legal framework of evidence‐informed priority setting Raise awareness on the importance of HTA among policymakers Include in overall HRH training, recruitment and retention strategy Foster collaborations between technology sponsors and providers and regulators

## 3 Recommendations

This policy brief puts forth the following recommendations to guide these efforts:

1. Leverage health taxes on alcohol, tobacco, sugar‐sweetened beverages and other products harmful to health to reduce the societal and healthcare costs and enhance long‐term sustainability in the health sector. Taking opportunity from the GST reform, (i) continue to place health taxes on the radar of the Ministry of Finance (MoF) and advocate for its periodic update and (ii) consider soft earmarking the health taxes to finance a broader array of public health programmes, particularly focusing on the promotion of healthy lifestyle, health screening and outreach public health activities. A targeted public awareness about the rationale behind health taxes and their impact on public health should be carried out to garner both policy level and public support.
2. Nurture and strengthen the capacity of HITAD under MoH. The HITAD should be empowered to facilitate effective decision‐making and resource allocation by conducting rigorous HTAs. Establishment of a dedicated multidisciplinary HTA committee to ensure comprehensive oversight of HTA nationwide is highly recommended. This committee will play a crucial role in reviewing and analysing HTA findings, providing expert recommendations and guiding informed decision‐making processes. Additionally, it is essential to raise awareness of the importance of HTA among policymakers and stakeholders.
3. Integrate HTA findings into policymaking and decision‐making processes within the healthcare system. Specifically, it is recommended to utilize HTA evidence for revising or updating the Health Service Standard (benefit package) and the National Essential Medicines List. HTA should also inform the development of clinical guidelines, procurement decisions and healthcare priority‐setting activities.

## 4 Conclusion

As highlighted earlier, free healthcare services in Bhutan are largely funded by the government through tax‐based financing and delivered free of cost at the point of care. Although this financing mechanism has contributed to improved health outcomes and ensured progress towards UHC, there are still a plethora of challenges to overcome. There is a pressing need to increase the fiscal space for health, considering the dwindling sources to the general fiscal space of the country. The need for healthcare will increase significantly owing to transitions in demography, epidemiology, economy and the ever‐changing demands of service users. To enhance the efficiency and quality of healthcare, it is crucial to reinforce a robust HTA framework now more than ever. By implementing HTA, Bhutan can ensure optimal resource allocation, prioritize interventions that offer the best value and make well‐informed decisions in line with the goals of UHC.

In conclusion, Bhutan must address the evolving healthcare landscape by expanding the fiscal space for health and strengthening the HTA framework. These measures will ensure efficient resource utilization, promote cost‐effective interventions and enable informed decision‐making, ultimately advancing the achievement of UHC.

## Author Contributions

U.T. conceptualized and designed the study, conducted literature search and drafted the manuscript. J.S. conducted literature search and revised the manuscript. D.T. and T.D. revised the manuscript.

## Ethics Statement

The authors have nothing to report.

## Consent

The authors have nothing to report.

## Conflicts of Interest

The authors declare no conflicts of interest.

## Consent for Publication

All co‐authors approved the publication of this article.

## Data Availability

Data sharing is not applicable to this article as no datasets were generated or analysed during the current study.
